# The WIRS motifs in Fat2 are required for *Drosophila* egg chamber rotation but not for elongation

**DOI:** 10.1242/dev.204201

**Published:** 2025-01-17

**Authors:** Akanksha Bhatt, Valentin Ruffine, Uwe Töpfer, Jinhee Ryu, Elisabeth Fischer-Friedrich, Christian Dahmann

**Affiliations:** ^1^ School of Science, Technische Universität Dresden, 01062 Dresden, Germany; ^2^ Cluster of Excellence Physics of Life, Technische Universität Dresden, 01062 Dresden, Germany

**Keywords:** *Drosophila*, Egg chamber, Basement membrane, Fat2, Collagen, Atomic force microscopy

## Abstract

The elongation of tissues and organs is important for proper morphogenesis in animal development. In *Drosophila* ovaries, the elongation of egg chambers involves aligned Collagen IV fiber-like structures, a gradient of extracellular matrix stiffness and actin-based protrusion-driven collective cell migration, leading to the rotation of the egg chamber. Egg chamber elongation and rotation depend on the atypical cadherin Fat2. Fat2 contains in its intracellular region three WRC interacting receptor sequence (WIRS) motifs, which previously had been shown to bind to the WAVE regulatory complex (WRC), a conserved actin regulator. Here, we show that in *fat2* mutant flies lacking the WIRS motifs, egg chambers fail to rotate and Collagen IV fiber-like structures are impaired, yet a gradient of extracellular matrix stiffness is established and egg chambers properly elongate. We conclude that the WIRS motifs are required for egg chamber rotation and that egg chamber rotation might be a prerequisite for proper formation of Collagen IV fiber-like structures. Egg chamber rotation, however, is dispensable for extracellular matrix stiffness gradient formation and for egg chamber elongation.

## Teaser

**Highlighted Article:** A sequence motif in the intracellular region of the atypical cadherin Fat2 is required for the rotation of *Drosophila* egg chambers, but is dispensable for egg chamber elongation.

## INTRODUCTION

**Fig. 1.**
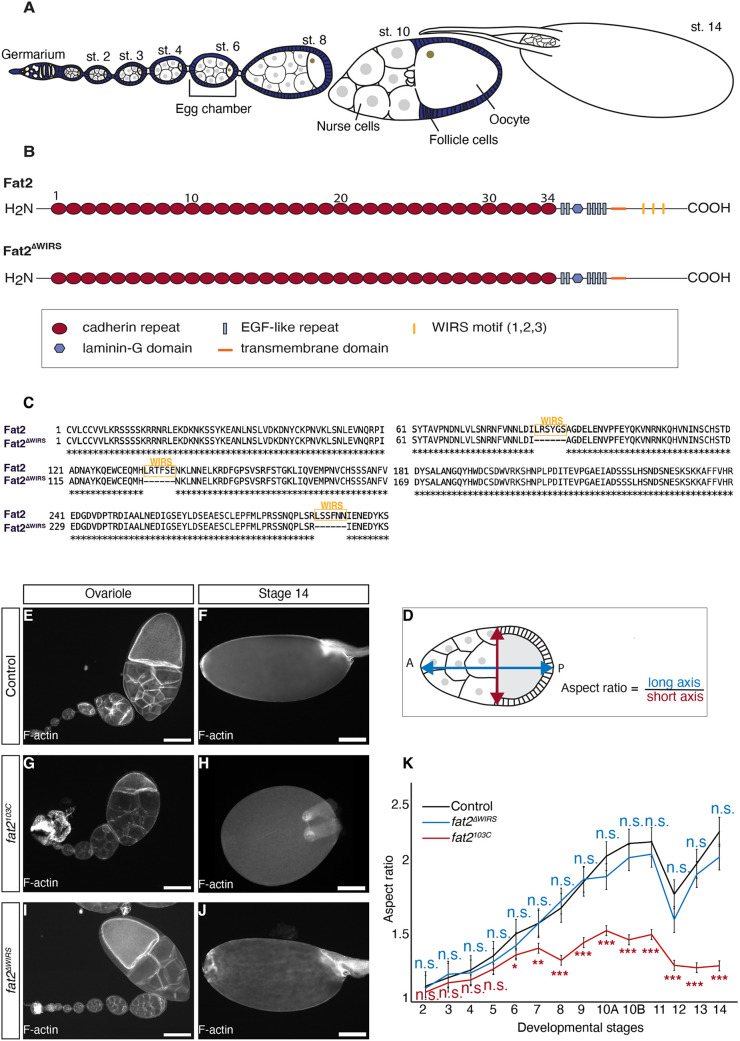
**WIRS motifs are dispensable for egg chamber elongation.** (A) Schematic of egg chamber development. (B) Domain structure of Fat2 and Fat2^ΔWIRS^. (C) Amino acid sequence alignment of part of the intracellular region of Fat2 and Fat2^ΔWIRS^. WIRS motifs are indicated. (D) Quantification of aspect ratio of egg chambers. (E-J) Ovarioles of indicated genotypes stained for F-actin. Scale bars: 100 µm. (K) Aspect ratio of egg chambers of indicated genotypes as a function of developmental stage. Data are mean±s.e.m. *n*>6 egg chambers for each genotype and stage. For comparisons of *fat2^ΔWIRS^* versus control (blue) and *fat2^ΔWIRS^* versus *fat2^103C^* (red): n.s., not significant; *P<0.05; **P<0.01; ***P<0.001 (unpaired Student's *t*-test).

The initial spherical symmetry of oocytes or early embryos is often broken through anisotropic growth to implement a body plan with a single long axis (Boutillon et al., 2024). In *Drosophila*, egg chambers, organs in which the oocyte matures, are initially spherical and, in the course of 7 days passing through 14 stages, grow in volume and elongate along their anteroposterior axis to give rise to mature eggs (Fig. 1A). Each egg chamber is composed of, in addition to the oocyte, 15 nurse cells and a surrounding sheet of epithelial follicle cells that is supported by a basement membrane (Spradling, 1993). Multiple processes drive egg chamber elongation, including, during stages 6-8, the formation of a ‘molecular corset’. The molecular corset restrains egg chamber expansion perpendicular to the anteroposterior axis and is composed of co-aligned actin filaments at the basal side of follicle cells and Collagen IV fiber-like structures within the basement membrane (Gutzeit et al., 1991; Haigo and Bilder, 2011). Egg chamber elongation during stages 2-8 coincides with the oriented collective migration of follicle cells, leading to the rotation of the egg chamber relative to its basement membrane along the anteroposterior axis (Cetera et al., 2014; Chen et al., 2016; Haigo and Bilder, 2011). Egg chamber rotation has been proposed to promote the alignment of actin filaments and Collagen IV fiber-like structures, and to contribute to egg chamber elongation (Cetera et al., 2014; Chen et al., 2016; Haigo and Bilder, 2011; Isabella and Horne-Badovinac, 2016; Williams et al., 2022). However, a recent study indicated that Collagen IV fiber-like structures can align and egg chambers can elongate in the absence of egg chamber rotation (Aurich and Dahmann, 2016).

The atypical cadherin Fat2 (also known as *kugelei*; Gutzeit et al., 1991) is required for egg chamber rotation, for tissue-level alignment of actin filaments and Collagen IV fiber-like structures, and for egg chamber elongation (Gutzeit et al., 1991; Viktorinova and Dahmann, 2013; Viktorinova et al., 2009). Fat2 is a transmembrane protein containing 34 cadherin repeats, six EGF-like repeats and one laminin G-like domain in its extracellular region, and three WRC interacting receptor sequence (WIRS) motifs in its intracellular region (Castillejo-Lopez et al., 2004; Chen et al., 2014; Squarr et al., 2016) (Fig. 1B). Fat2 localizes at the lagging end of migrating follicle cells, where it is required *in trans* to enrich the receptor tyrosine phosphatase Lar and the WAVE regulatory complex (WRC) to the leading edge of the following cells (Barlan et al., 2017; Viktorinova and Dahmann, 2013; Williams et al., 2022). The WRC is composed of the five components: WAVE, Sra1 (also known as Cyfip1), Nap1 (also known as Hem-2), Abi and HSPC300 (Chen et al., 2010). The WRC promotes actin nucleation activity of Actin-relating proteins 2/3 (Arp2/3) (Bieling and Rottner, 2023), thereby resulting in formation of protrusions on the leading edge of follicle cells (Williams et al., 2022). Fat2 also interacts with the WRC *in cis*. In a GST pull-down assay, Abi binds to the intracellular region of Fat2, but not to a mutant form lacking the WIRS motifs (Squarr et al., 2016). Moreover, female flies expressing a mutant form of Abi lacking the conserved WIRS-binding surface generated with low penetrance (∼20%) round egg chambers that failed to rotate and no longer displayed oriented protrusions on follicle cells, indicating that binding of Fat2 to the WRC *in cis* plays a role in egg chamber rotation and elongation (Squarr et al., 2016). However, the *in vivo* role of the three WIRS motifs of the intracellular region of Fat2 remained unclear.

Here, we have used CRISPR/Cas9 to generate mutant flies lacking the three WIRS motifs within the Fat2 intracellular region. Our analyses suggest that the WIRS motifs within the intracellular region of Fat2 are dispensable for egg chamber elongation, for proper tissue-level alignment of actin filaments and Collagen IV fiber-like structures, and for stiffness gradient formation in the basement membrane, but are essential for proper orientation of protrusions on follicle cells and for egg chamber rotation. These observations uncouple egg chamber elongation from egg chamber rotation and provide *in vivo* evidence that Fat2 also interacts with the WRC *in cis* to direct follicle cell migration.

## RESULTS AND DISCUSSION

### WIRS motifs are dispensable for egg chamber elongation

The intracellular region of Fat2 contains three WIRS motifs that previously had been shown to interact biochemically with components of the WRC (Squarr et al., 2016). To test the *in vivo* role of the WIRS motifs, we used CRISPR/Cas9 to modify the coding capacity of the *fat2* locus such that the three amino acid sequences that constitute the three WIRS motifs are deleted (Fig. 1B,C) (see Materials and Methods). We refer to this allele as *fat2^ΔWIRS^*. Flies homozygous for *fat2^ΔWIRS^* were viable and fertile.

We first tested whether the three WIRS motifs within the intracellular region of Fat2 were required for egg chamber elongation. We quantified the extent of egg chamber elongation by calculating the aspect ratio of long axis versus short axis of egg chambers from stage 2 to 14 (Fig. 1D). Egg chambers from control flies elongated from an aspect ratio of ∼1 at stage 2 to a final aspect ratio of ∼2.3 at stage 14 (Fig. 1E,F,K). Consistent with previous reports (Aurich and Dahmann, 2016; Viktorinova et al., 2009), egg chambers from flies homozygous for a null allele of *fat2*, *fat2^103C^*, failed to properly elongate (Fig. 1G,H), reaching an aspect ratio of ∼1.3 at stage 14 (Fig. 1K). Egg chambers from homozygous *fat2^ΔWIRS^* flies elongated indistinguishably from control egg chambers (Fig. 1I-K). We conclude that the three WIRS motifs within the intracellular region of Fat2 are dispensable for egg chamber elongation.

### WIRS motifs are dispensable for alignment of actin filaments and Collagen IV fiber-like structures

**Fig. 2.**
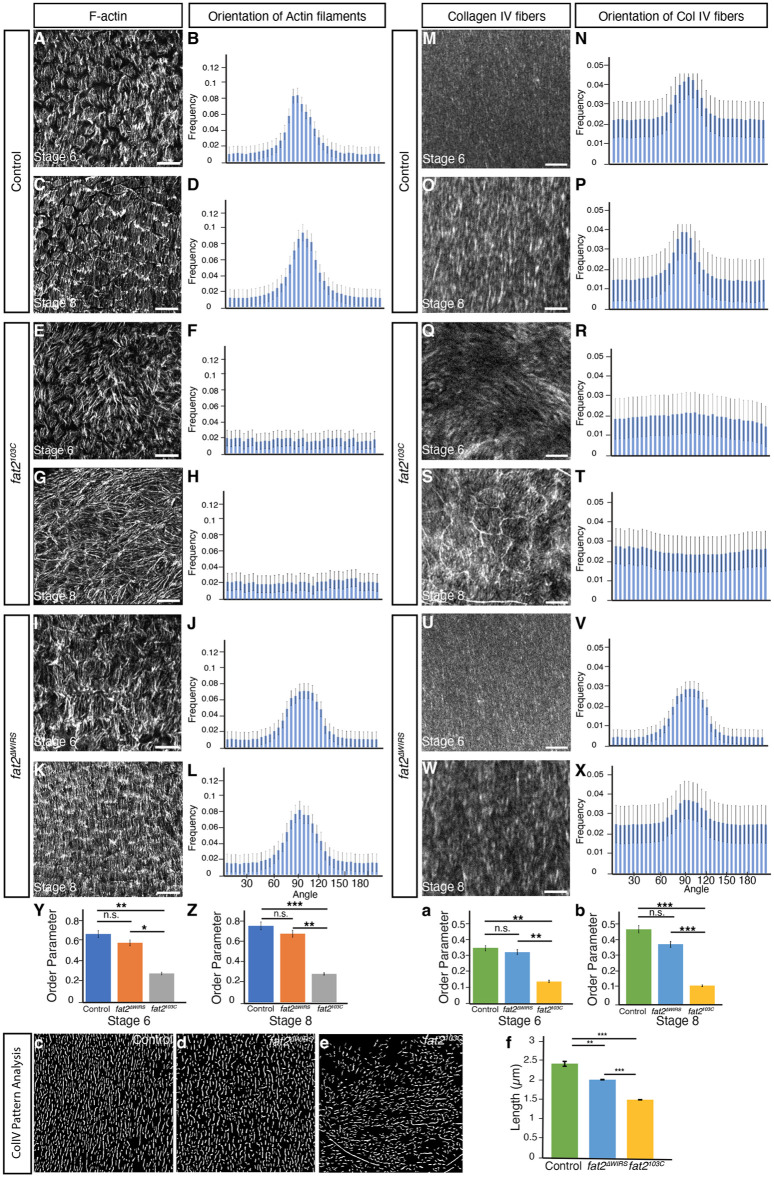
**WIRS motifs are dispensable for the alignment of actin filaments and Collagen IV fiber-like structures.** (A,C,E,G,I,K) Egg chambers of indicated genotypes and stages stained for F-actin. Scale bars: 10 µm. (B,D,F,H,J,L) Frequency distribution of orientation of actin filaments for indicated genotypes and stages. 0° and 180° correspond to the anteroposterior axis. Data are mean±s.e.m. *n*>5 egg chambers for each genotype and stage. (M,O,Q,S,U,W) Egg chambers of indicated genotypes and stages stained for Collagen IV-GFP. Scale bars: 10 µm. (N,P,R,T,V,X) Frequency distribution of orientation of Collagen IV-GFP fiber-like structures for indicated genotypes and stages. 0° and 180° correspond to anteroposterior axis. Data are mean±s.e.m. *n*=5 egg chambers for each genotype and stage. (Y-b) Order parameters describing alignment of F-actin filaments (Y,Z) or Collagen IV fiber-like structures (a,b) relative to anteroposterior axis of egg chambers of indicated stage and genotype. Data are mean±s.e.m. *n*>5 egg chambers for each genotype and stage. n.s., not significant; *P<0.05; **P<0.01; ***P<0.001 (unpaired Student's *t*-test). (c-e) Maps of Collagen IV fiber-like structures of central region of stage 8 egg chambers of indicated genotypes. (f) Length of Collagen IV fiber-like structures of central region of stage 8 egg chambers of indicated genotypes. Data are mean±s.e.m. *n*>5 egg chambers for each genotype and stage. **P<0.01, ***P<0.001 (unpaired Student's *t*-test).

Follicle cells display actin filaments and Collagen IV fiber-like structures, which are aligned perpendicular to the (future) long axis of the egg chamber (Cetera et al., 2014; Gutzeit, 1990; Haigo and Bilder, 2011). To test whether the three WIRS motifs within the intracellular region of Fat2 are required for these tissue-level alignments, we visualized the fiber-like structures using Collagen IV-GFP (encoded by *vkg-GFP*), a protein trap in the *Collagen IV α2* gene (Buszczak et al., 2007), and filamentous actin (F-actin) by phalloidin staining. We calculated the order parameter S_AP_ (Cetera et al., 2014), which characterizes the alignment of structures relative to the anteroposterior axis of egg chambers. S_AP_ can vary from +1 (alignment perpendicular to anteroposterior axis) to −1 (alignment parallel to anteroposterior axis). In egg chambers of homozygous *fat2^ΔWIRS^* mutant flies, actin filaments were aligned perpendicular to the anteroposterior axis at stages 6 and 8, similar to controls (Fig. 2A-D,I-L,Y,Z), whereas in *fat2^103C^* homozygous mutant flies, actin filaments were locally aligned, yet failed to be aligned perpendicular the anteroposterior axis (Fig. 2E-H,Y,Z), as reported previously (Aurich and Dahmann, 2016; Gutzeit et al., 1991; Viktorinova and Dahmann, 2013; Viktorinova et al., 2009). Collagen IV fiber-like structures were aligned perpendicular to the anteroposterior axis in egg chambers of homozygous *fat2^ΔWIRS^* mutant flies and of controls (Fig. 2M-P,U-X,a,b), whereas no such alignment was detected in egg chambers of *fat2^103C^* mutant flies (Fig. 2Q-T,a,b), consistent with a previous report (Aurich and Dahmann, 2016). However, these fiber-like structures were shorter in *fat2^ΔWIRS^* mutants compared with controls (Fig. 2c-f) (see Materials and Methods). Thus, while the WIRS motifs within the intracellular region of Fat2 are dispensable for the tissue-level alignment of actin filaments and Collagen IV fiber-like structures, they are required to form properly sized Collagen IV fiber-like structures.

### Collagen IV fiber-like structures are embedded within the basement membrane and are regions of higher stiffness

**Fig. 3.**
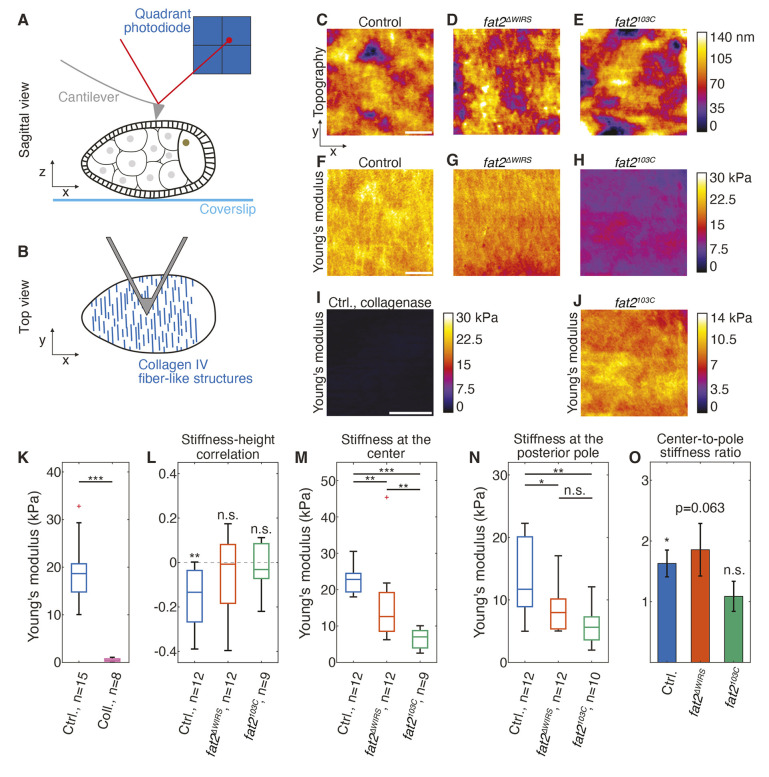
**Topography and stiffness maps of basement membrane.** (A) Schematic sagittal view of atomic force microscopy (AFM) measurement on the egg chamber. Indentations are performed in the basement membrane, close to center of the egg chamber. (B) Schematic top view of AFM cantilever on measured egg chamber. Cantilever orientation is orthogonal to the long axis of the egg chamber and dominant orientation of Collagen IV fiber-like structures is thus orthogonal to the fast axis when scanning (*x*-axis). (C-E) Topography maps measured in the center region of basement membrane of stage 8 egg chambers of indicated genotypes. Scale bar: 5 µm. (F-H) Stiffness maps of regions shown in C-E, respectively. (I) Stiffness map measured in center region of a stage 8 control egg chamber treated with collagenase. Scale bar: 2 µm. (J) The same stiffness map as in H, with adapted color scale. (K) Apparent Young's moduli of the center region of the basement membrane in stage 8 egg chambers from control flies measured in control condition or after collagenase treatment (Coll.). ***P<0.001 (Wilcoxon rank sum test). (L) Correlation coefficients between stiffness maps and topography maps measured in the central region of stage 8 egg chambers of the indicated genotypes. n.s., not significant; **P<0.01 (one-sample *t*-test for a zero mean). (M,N) Apparent Young's moduli measured in the central region (M) or at the posterior pole region (N) of stage 8 egg chambers of the indicated genotypes. n.s., not significant; *P<0.05; **P<0.01; ***P<0.001 (Wilcoxon rank sum test). (O) Ratio between mean Young's moduli in the center region and mean Young's moduli at the posterior pole, for each genotype. Error bars denote one standard error of the mean ratio. n.s., not significant; *P<0.05 (see Materials and Methods for a description of the statistical test). Box and whisker plots indicate the median (horizontal line), the IQR from the 25th to 75th percentile (box), and minimum and maximum values (whiskers).

Collagen IV contributes to the stiffness of the basement membrane of egg chambers (Chlasta et al., 2017; Crest et al., 2017; Töpfer et al., 2022). Using atomic force microscopy (AFM) to probe the topography and stiffness of decellularized basement membranes of egg chambers, Chlasta et al. reported protruding structures of increased stiffness resembling Collagen IV fiber-like structures (Chlasta et al., 2017). We revisited the topography and stiffness of the basement membrane in living egg chambers. To this end, we used AFM in contact mode to obtain at the same time topographical (height) and stiffness maps of 20 µm×20 µm regions of the basement membrane of stage 8 egg chambers (Fig. 3A,B; see Materials and Methods). The topography of control egg chambers was seemingly complex and topographical characteristics resembling Collagen IV fiber-like structures were not apparent (Fig. 3C). Fourier analysis along the anteroposterior axis (see Materials and Methods) showed that the topography of egg chambers of *fat2^103C^* or *fat2^ΔWIRS^* homozygous mutant flies was smoother compared with the topography of control egg chambers (Fig. 3D,E, Fig. S1A), indicating differences in the structure of the basement membranes. The topography negatively correlated with the spatial pattern of stiffness in control egg chambers (Fig. 3C,F,L), suggesting the presence of soft elevated structures. However, stiffness and topography did not correlate in egg chambers of *fat2^103C^* or *fat2^ΔWIRS^* homozygous mutant flies (Fig. 3D,E,G,H,L), indicating that such soft elevated structures are not present in the basement membrane of these mutants. The median basement membrane stiffness (effective Young's modulus) of control egg chambers was ∼20 kPa (Fig. 3F,K,M). Strikingly, stripes of increased stiffness (∼10% higher than bulk) were detectable (Fig. 3F). These stripes had a size comparable with the size of Collagen IV fiber-like structures detected by confocal microscopy (compare Figs 2O and 3F) and were oriented perpendicular to the long axis of the egg chamber (Fig. 3F). Prior treatment of control egg chambers with Collagenase, which degrades basement membrane components (Töpfer et al., 2022), resulted in a significant reduction of stiffness (Fig. 3I,K). Stripes of increased stiffness were no longer detectable (Fig. 3I). Taken together, elevated structures of the basement membrane tend to have lower stiffness. Moreover, Collagen IV fiber-like structures are embedded within the basement membrane and are regions of higher stiffness.

### WIRS motifs are required for proper basement membrane stiffness

To test whether the three WIRS motifs of the intracellular region of Fat2 are required for proper basement membrane stiffness in the bulk or in the stripes, we further analyzed basement membrane stiffness in the center of stage 8 *fat2^ΔWIRS^* egg chambers. Compared with controls, the basement membrane stiffness in the bulk was reduced by ∼45% in egg chambers of *fat2^ΔWIRS^* homozygous mutant flies (Fig. 3F,G,M). Stripes of increased stiffness were still detectable (Fig. 3G). The stiffness of the stripes compared to the bulk was increased by ∼7%, similar to the control (Fig. 3F,G). Moreover, these stripes of increased stiffness were oriented perpendicular to the long axis of the egg chamber, but appeared to be shorter compared with the stripes in control egg chambers (Fig. 3F,G). For comparison, the basement membrane stiffness of egg chambers of *fat2^103C^* homozygous mutant flies was reduced by 70%, with a median stiffness of ∼7 kPa (Fig. 3H,J,M). Stripes of increased stiffness were not detectable (Fig. 3H,J). Thus, the WIRS motifs of Fat2 are required for proper basement membrane stiffness.

### WIRS motifs are dispensable for a gradient of basement membrane stiffness

The stiffness of the basement membrane is graded, displaying a higher stiffness in the central region compared with the pole region of the egg chamber (Crest et al., 2017; Töpfer et al., 2022), which has been proposed to contribute to egg chamber elongation (Crest et al., 2017). Using AFM, we next tested whether the three WIRS motifs of the intracellular region of Fat2 are required to establish this stiffness gradient. While basement membrane stiffness was lower in both central region and posterior pole region of stage 8 egg chambers from *fat2^ΔWIRS^* homozygous mutant flies compared with controls, basement membrane stiffness was still higher at the central region compared with the pole region (compare Fig. 3M with Fig. 3N,O). By contrast, in egg chambers of *fat2^103C^* homozygous mutant flies, basement membrane stiffness was significantly lower both in the center region and the posterior pole region, and the stiffness was similar in both regions (compare Fig. 3M with Fig. 3N,O). Thus, the WIRS motifs of Fat2 are dispensable for the establishment of the basement membrane stiffness gradient.

### WIRS motifs are required for egg chamber rotation

**Fig. 4.**
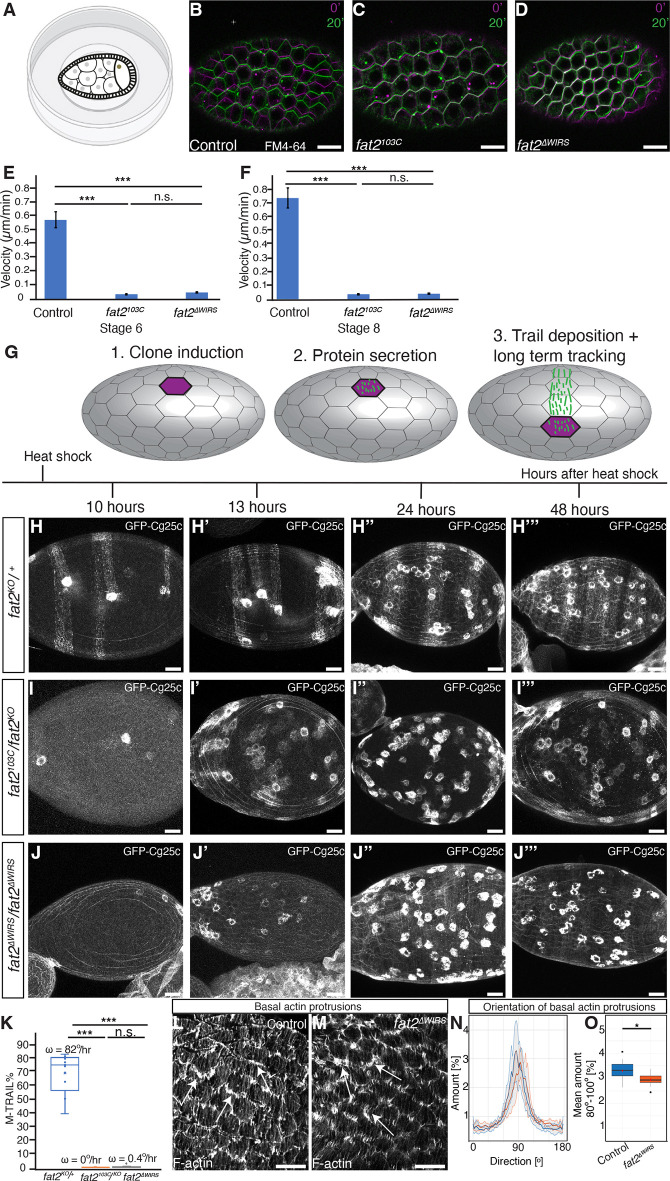
**WIRS motifs are required for egg chamber rotation.** (A) Schematic of analyzing rotation of *ex vivo* cultured egg chambers. (B-D) Overlays of egg chambers of the indicated genotypes and stages shown at 0 min (magenta) and 20 min (green) of incubation *ex vivo*. Cell membranes are visualized using FM4-64. Scale bars: 10 µm. Images are also shown in Fig. S2 alongside separate images for each time point. (E,F) Velocity of rotation of stage 6 (E) or stage 8 (F) egg chambers of the indicated genotypes. Data are mean±s.e.m. *n*>5 egg chambers for each genotype and stage. n.s., not significant; ***P<0.001 (unpaired Student's *t*-test). (G) Schematic of M-TRAIL assay. (H-J‴) Stage 8-9 egg chambers of the indicated genotypes at the indicated time after heat shock to induce cell clones expressing GFP-Cg25C. Scale bars: 20 µm. (K) Box plots of the ratio of length of GFP-Cg25c trails and circumference of egg chamber in percent. *n*=10 egg chambers for each genotype and stage. n.s., not significant; ***P<0.001 (unpaired Student's *t*-test). Means of angular velocity of egg chambers are also indicated. Box and whisker plot indicates the median (horizontal line), the IQR from the 25th to 75th percentile (box), and minimum and maximum values (whiskers). (L,M) Stage 8 egg chambers of indicated genotypes stained for F-actin. Arrows indicate cell protrusions. Scale bars: 10 µm. (N,O) Mean orientation of actin-rich protrusions (N) and the mean amount of actin-rich protrusions oriented between 80° and 100° (O) relative to the anteroposterior axis for the indicated genotypes. The color code for the genotypes is identical for N and O. *n*=7 egg chambers per genotype. *P<0.05 (unpaired Student's *t*-test).

Follicle cells display F-actin-rich cellular protrusions at their leading edge that result in collective cell migration and thereby egg chamber rotation during stages 1-8 (Cetera et al., 2014; Squarr et al., 2016). Egg chamber rotation has been proposed to be necessary for egg chamber elongation (Bilder and Haigo, 2012; Chen et al., 2017; Haigo and Bilder, 2011). To test whether the WIRS motifs of the intracellular region of Fat2 are required for egg chamber rotation, we used two approaches. First, we dissected ovaries, cultured them *ex vivo* and used time-lapse microscopy to observe whether stage 6 or stage 8 egg chambers rotated (Fig. 4A). Control stage 6 and stage 8 egg chambers rotated with a mean velocity of ∼0.6 µm/s and ∼0.7 µm/s, respectively (Fig. 4B,E,F, Fig. S2) (Movie 1), consistent with a previous publication (Aurich and Dahmann, 2016). Egg chambers of *fat2^103C^* mutant or *fat2^ΔWIRS^* mutant flies did not noticeably rotate (Fig. 4C-F, Fig. S2) (Movie 1).

Second, we applied the matrix-labeling technique for real-time and inferred location (M-TRAIL) to measure egg chamber rotation velocity *in vivo*. This assay relies on the FRT-FLP-mediated generation of cell clones that secrete a GFP-tagged version of Collagen IV1α (GFP-Cg25c) (Chen et al., 2017) (Fig. 4G). Since the egg chamber rotates relative to its own basement membrane, the secreted GFP-Cg25c forms a trail within the basement membrane. The angular velocity of trail deposition is a measure of egg chamber rotation that takes place between the beginning of GFP-Cg25c secretion upon clone induction and the time of analysis. We generated clones secreting GFP-Cg25c and analyzed stage 9 egg chambers 10 h, 13 h, 24 h or 48 h later for the presence of trails. Based on the duration of egg chamber development (Spradling, 1993) and the time required for GFP-Cg25c secretion (Chen et al., 2017), we expected that the analysis of egg chambers 48 h after clone induction would measure rotation from stage 2-3 onwards. We calculated the angular velocity of egg chamber rotation based on trails deposited 10 h after clone generation. In control egg chambers, we identified GFP-Cg25c trails at all time points analyzed (Fig. 4H-H‴). The calculated angular velocity was ∼82°/h (Fig. 4K), consistent with a previous report (Chen et al., 2017). No trails were detected for egg chambers of *fat2^103C^/fat2^KO^* mutant flies (Fig. 4I-I‴,K), consistent with a previous report (Chen et al., 2017). Likewise, no trails were detected, even 48 h after clone induction, for egg chambers of *fat2^ΔWIRS^* homozygous mutant flies (Fig. 4J-J‴,K). The measured angular velocity of *fat2^103C^/fat2^KO^* or *fat2^ΔWIRS^* homozygous mutant flies was highly decreased compared with controls (Fig. 4K). Consistent with the lack of observable egg chamber rotation, basal actin-rich protrusions of follicle cells were not properly oriented in follicle cells of *fat2^ΔWIRS^* homozygous mutant egg chambers compared with controls (Fig. 4L-O). We conclude that the WIRS motifs of the intracellular region of Fat2 are required for egg chamber rotation.

In summary, the three WIRS motifs within the intracellular region of Fat2 are required for egg chamber rotation, but are dispensable for establishing aligned actin filaments and Collagen IV fiber-like structures, for establishing a gradient of basement membrane stiffness and for egg chamber elongation. A mutant form lacking the entire intracellular region of Fat2 results in similar phenotypic alterations (Aurich and Dahmann, 2016; Cerqueira Campos et al., 2020; Chen et al., 2017), indicating that the WIRS motifs are the main functionally important sequences within the intracellular region of Fat2. A notable exception is that, as assayed by M-TRAIL, deletion of the entire intracellular region of Fat2 results in a slowdown of egg chamber rotation (Chen et al., 2017), whereas deletion of the three WIRS motifs results in no observable rotation (Fig. 4). The reason for the different behaviors of egg chambers expressing these two mutant forms of Fat2 in the M-TRAIL assay is unknown. However, in an *ex vivo* assay, rotation of egg chambers was observed neither when the entire intracellular region of Fat2 (Aurich and Dahmann, 2016; Chen et al., 2017) nor when the three WIRS motifs (Fig. 4) were deleted. How actin filaments and Collagen IV fiber-like structures are aligned in the absence of egg chamber rotation needs to be revealed in the future. The failure of egg chambers of *fat2^ΔWIRS^* homozygous mutant flies to rotate, yet their capacity to properly elongate (Fig. 1), indicates that egg chamber rotation and egg chamber elongation are separable processes.

Previous work showed that Fat2 directs follicle cell migration and egg chamber rotation by stabilizing the WAVE complex *in trans* (Barlan et al., 2017; Williams et al., 2022). Moreover, the presence of three WIRS motifs in the intracellular region of Fat2 and the interaction of the WRC component Abi with Fat2 in a GST pull-down assay also suggest that Fat2 interacts with the WAVE complex *in cis* (Squarr et al., 2016). Our work now provides *in vivo* evidence that Fat2 acts *in cis* with the WAVE complex to direct follicle cell migration.

## MATERIALS AND METHODS

### Fly stocks and genetics

The following fly stocks were used: *fat2^103C^* (Viktorinova et al., 2009), *vkg-*GFP (Buszczak et al., 2007) and *Act5C>CD2>Gal4; UAS-GFP-Cg25C/CyO; fat2^KO^/TM6C* (Chen et al., 2017). Control flies were *y w.* Flies were raised at 25**°**C on standard food.

The genotypes of the adult flies were as follows:

Fig. 1: (E,F) *y w*; (G,H) *fat2^103C^/fat2^103C^*; (I,J) *fat2^ΔWIRS^/fat2^ΔWIRS^*.

Fig. 2: (A,C) *y w*; (E,G) *fat2^103C^/fat2^103C^*; (I,K) *fat2^ΔWIRS^/fat2^ΔWIRS^*; (M,O,c) *vkg-GFP/CyO*; (Q,S,e) *y w; vkg-GFP/CyO; fat2^103C^/fat2^103C^*; (U,W,d) *y w; vkg-GFP/CyO; fat2^ΔWIRS^/fat2^ΔWIRS^*.

Fig. 3: (C,F) *vkg-GFP/CyO*; (D,G) *fat2^ΔWIRS^/ fat2^ΔWIRS^*; (E,H) *fat2^103C^/fat2^103C^*.

Fig. 4: (B) *y w*; (C) *fat2^103C^/fat2^103C^*; (D) *fat2^ΔWIRS^/ fat2^ΔWIRS^*; (H-H‴) *y w hsp-flp/Act5C>CD2>Gal4; UAS-GFP-Cg25c/CyO; fat2^KO^/+*; (I-I‴) *y w hsp-flp/Act5C>CD2>Gal4; UAS-GFP-Cg25c/CyO; fat2^103C^/fat2^KO^*; (J-J‴) *y w hsp-flp/Act5C>CD2>Gal4; UAS-GFP-Cg25c/CyO; fat2^ΔWIRS^/ fat2^ΔWIRS^*; (L) *y w*; (M) *fat2^ΔWIRS^/fat2^ΔWIRS^*.

### Generation of *fat2^ΔWIRS^* flies

A single gRNA was designed with E-CRISP (Heigwer et al., 2014) and cloned in pCDF5 (Addgene, 73914) (Port and Bullock, 2016). pBSK II+(Alting-Mees and Short, 1989) was used as a donor vector. Genomic DNA from *y w* control flies were used to generate 5 PCR fragments. Oligonucleotides (Table S1) were modified to introduce a silent mutation in the PAM motif, delete the three WIRS motifs, enable restriction enzyme digestion with BamHI, and include a 795 bp left homology arm and a 1703 bp right homology arm. Deletion of the WIRS motifs was introduced by designing oligonucleotides with overhangs complementary to the neighboring fragment but skipping the WIRS motifs. PCR fragments were assembled with Gibson assembly (Invitrogen, A46627), and cloned into the donor vector with restriction enzyme digestion and subsequent ligation. The resulting flies were validated by DNA sequencing.

### Immunostainings

Ovaries from 2- to 3-day-old overfed flies were dissected in 1×PBS followed by fixation in 4% paraformaldehyde and 0.1% Triton X-100 for 20 min. Immunostainings followed standard protocols. Primary antibody used was rabbit anti-GFP (Clontech, 632592, 1:2000). Secondary antibody was goat anti-rabbit Alexa 488 (Molecular Probes, A27034, 1:200). Rhodamine-phalloidin (Thermo Fisher Scientific, R415, 1:200) was used at a dilution of 1:200. Images were acquired using a Zeiss LSM 980 laser scanning microscope.

### Aspect ratio

Egg chamber stages were determined as previously described (Prasad et al., 2007; Spradling, 1993). The length and width of the egg chambers was determined using Fiji (Schindelin et al., 2012).

### Quantification of the orientation of actin filaments and Collagen IV fiber-like structures

To measure the orientation of actin filaments and Collagen IV fiber-like structures, we used the Directionality tool in Fiji (Schindelin et al., 2012).

### Quantification of the length of Collagen IV fiber-like structures

Pattern analysis and quantification of Collagen IV fiber-like structures was carried out as previously published (Ku et al., 2023).

### Quantification of the orientation of basal actin-rich protrusions

The orientation of the basal actin-rich protrusions was quantified as previously published (Töpfer et al., 2024).

### Egg chamber rotation

Egg chambers were dissected in Schneider's medium cocktail (Prasad et al., 2007) and adhered to poly-D-lysine-coated (Thermo Fisher Scientific A38904-01) glass bottom microwell dishes (MatTek P35G-1.5-20-C). Egg chambers were imaged for 20 min. FM4-64 (Molecular Probes, T-3166, 1.6 µM) dye was used to visualize cell membranes.

M-TRAIL was conducted as previously published (Chen et al., 2017). Follicle cell clones were induced by exposing 2- to 3-day-old overfed flies for 5 min to 37°C using a water bath. Ovaries were then dissected and stained after the indicated times after heat shock. The egg chambers were then dissected and fixed using standard protocols.

### AFM measurements

The AFM force mapping of the basement membrane of egg chambers was performed with a JPK Nanowizard 4XP AFM (Bruker) mounted on an LSM 700 confocal microscope (Zeiss). The probes used were cantilevers with quadratic pyramidal tips (MLCT-BIO-D, Bruker) with a nominal spring constant of 0.03 N/m and a half-angle to face of 35**°**. The actual spring constant of the cantilever was measured before each experiment, using the thermal fluctuation method (JPK SPM software, Bruker). Measurements were carried out on stage 8 egg chambers in Schneider's medium cocktail (Prasad et al., 2007) at room temperature. When indicated, 0.05% w/v collagenase type I (Merck, SCR103) was added 30 min before the measurements. The measured egg chambers were oriented so that their anteroposterior axis was approximately aligned with the fast axis of the AFM scan. The fast axis is represented horizontally in the topography and stiffness maps (Fig. 3C-J, *x*-axis) and is perpendicular to the cantilever (Fig. 3B). Measurements were taken in the QI™ Advanced mode (Bruker) with a setpoint force of 0.6 nN and an indentation speed of 100 µm/s. A square area of 5×5 to 20×20 µm^2^ was selected close to the center of the egg chamber and divided into a grid of 128×128 or 180×180 points. Indentation of the sample was then automatically performed, yielding one indentation curve for each point. We note that the differences in cantilever tip shape and indentation speed between this study and previous similar works (Chlasta et al., 2017; Crest et al., 2017; Töpfer et al., 2022) can account for the differences in the measured effective Young's moduli (Fig. S1B). Dish preparation and mounting of egg chambers were performed as described previously (Töpfer et al., 2022).

### Analysis of AFM data

Initial processing of the AFM data was carried out with the JPK Data Processing application (Bruker). The indentation curves were first processed individually. For the study of the basement membrane topography, the vertical position of the surface of the sample was defined as the cantilever tip position at which the force reached 20% of the setpoint force. To generate maps of effective Young's moduli (stiffness), the Hertz-Sneddon model was adapted to the tip geometry of the cantilever and, assuming a Poisson ratio of 0.5, was fitted onto the indentation curves. The fitting range included the force curves up to a maximum force of 500 pN, corresponding to an indentation depth of ∼200 nm. This fit procedure yielded Young's moduli with similar values and identical trends upon perturbations compared to the ones obtained when limiting the fitting range to a 50 nm indentation depth (Fig. S1C). At the same time, fitting up to 500 pN gave fit results with less variability. The obtained topography maps and stiffness maps were first processed within the JPK data processing application. The topography maps were corrected by applying 3rd order plane fitting and 1st order line levelling. Furthermore, smoothing of topography and stiffness maps was carried out using a median filter and a Savitzky-Golay filter.

For stiffness comparisons across experimental conditions, the stiffness median for a 5×5 µm² square was calculated. For measurements of stiffness at the posterior pole, a largely flat region was selected.

### Fourier transform analysis of topography maps

Topography maps were cropped to remove large defects or artefacts when present. Then, the discrete two-dimensional Fourier transform of each map was calculated using MATLAB. With *F* an *N*×*N* matrix (e.g. a topography map), its two-dimensional discrete Fourier transform is 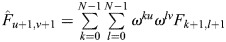, where *ω*=*e*^−2*πi*/*N*^ and 0≤*u*, *v*≤*N*−1.

For each map, the Fourier transform profile in the x-direction was defined as 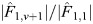, with 0≤*v*≤*N*−1. Then, the profiles were pooled by genotype and the average profiles were plotted as a function of the inverse wavelength 1/*λ*_x_=*v*/*L*, where *L* is the size of the map. As the values of inverse wavelength at which each of these discrete profiles is defined depend on the size of the map, profiles calculated from maps smaller than 20×20 µm^2^ were interpolated before pooling to obtain equivalent Fourier coefficients that could be pooled with the true Fourier coefficients of maps of that size.

### Statistical analysis on center-to-pole stiffness ratios

For each genotype, the mean center-to-pole stiffness ratio was estimated as the ratio 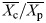 between the mean stiffness measured at the center and the mean stiffness measured at the posterior pole of egg chambers. The standard error of the mean of the ratio was calculated by error propagation as:

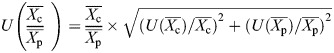
where 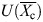 [respectively 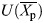] is the standard error of the mean of the stiffness at the center (respectively at the pole). The significance of the difference from 1 of the mean stiffness ratio was then evaluated using an unpaired Student's *t*-test, as follows. The test statistic was calculated as 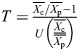. The effective degrees of freedom were estimated with the Welch-Satterthwaite equation (Lovric, 2011):

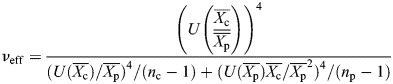
where *n*_c_ (respectively *n*_p_) is the sample size of the measurements of stiffness at the center (respectively at the pole). Finally, the *P*-value was calculated assuming a two-tailed *t*-distribution with *ν*_eff_ degrees of freedom for *T*.

### Statistical analysis

Statistical significance was tested using an unpaired Student's *t*-test using the R package or the Wilcoxon rank sum test using MATLAB. The number of replicates refers to egg chambers isolated from different individual flies.

## Supplementary Material



10.1242/develop.204201_sup1 Supplementary information
