# Supplementary material for: The WIRS motifs in Fat2 are required for Drosophila egg chamber rotation but not for elongation
Source: Development. 2025 Jan 17;152(2):DEV204201. doi: 10.1242/dev.204201 (PMC11829772; doi:10.1242/dev.204201)
Supplement: Supplementary information [file develop-152-204201-s1.pdf]

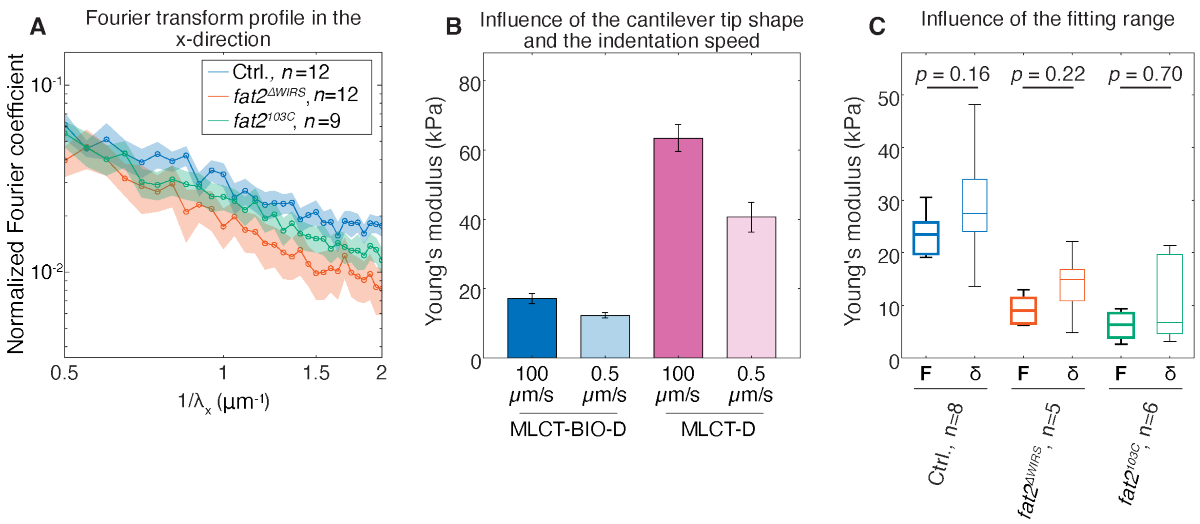

Fig. S1. Fourier analysis of topography maps, influence of the AFM cantilever tip shape, the indentation speed and the fitting range on the measured effective Young's modulus.

(A) Population-averaged profiles in the x-direction of the 2-D discrete Fourier transform of topography maps acquired in the central region of stage 8 egg chambers of the indicated genotypes. Stripes oriented perpendicular to the anteroposterior axis of the egg chamber would result in peaks in the x-direction profile. The represented x-axis variable is the inverse of the length scale corresponding to the Fourier coefficients, see Materials and Methods. Shaded areas represent one standard error of the mean.

(B) Comparison of the effective Young's moduli obtained by indenting the center region of one stage 8 egg chamber from a control fly with two different AFM cantilever models and two different indentation speeds. MLCT cantilevers were used in similar experiments by Crest et al. and Töpfer et al. with an indentation speed of 0.4-0.5 μm/s (Crest et al., 2017; Töpfer et al., 2022). The quadratic pyramid tips of MLCT cantilevers have a narrower half-angle to face (12.6°) than the tips of MLCT-BIO cantilevers (35°). Error bars show one standard deviation.

(C) Comparison of the effective Young's moduli obtained through two fitting methods for one dataset of measurements in the center region. The fitting range is either limited to forces below 500 pN (thick lines and bold "F"), or to indentation depths below 50 nm (thin lines and "δ"). The former method was used throughout this study, while the latter was used in previous works (Chlasta et al., 2017; Crest et al., 2017; Töpfer et al., 2022). For each genotype, the choice of fitting method gave similar values and preserved trends (Wilcoxon rank sum test).

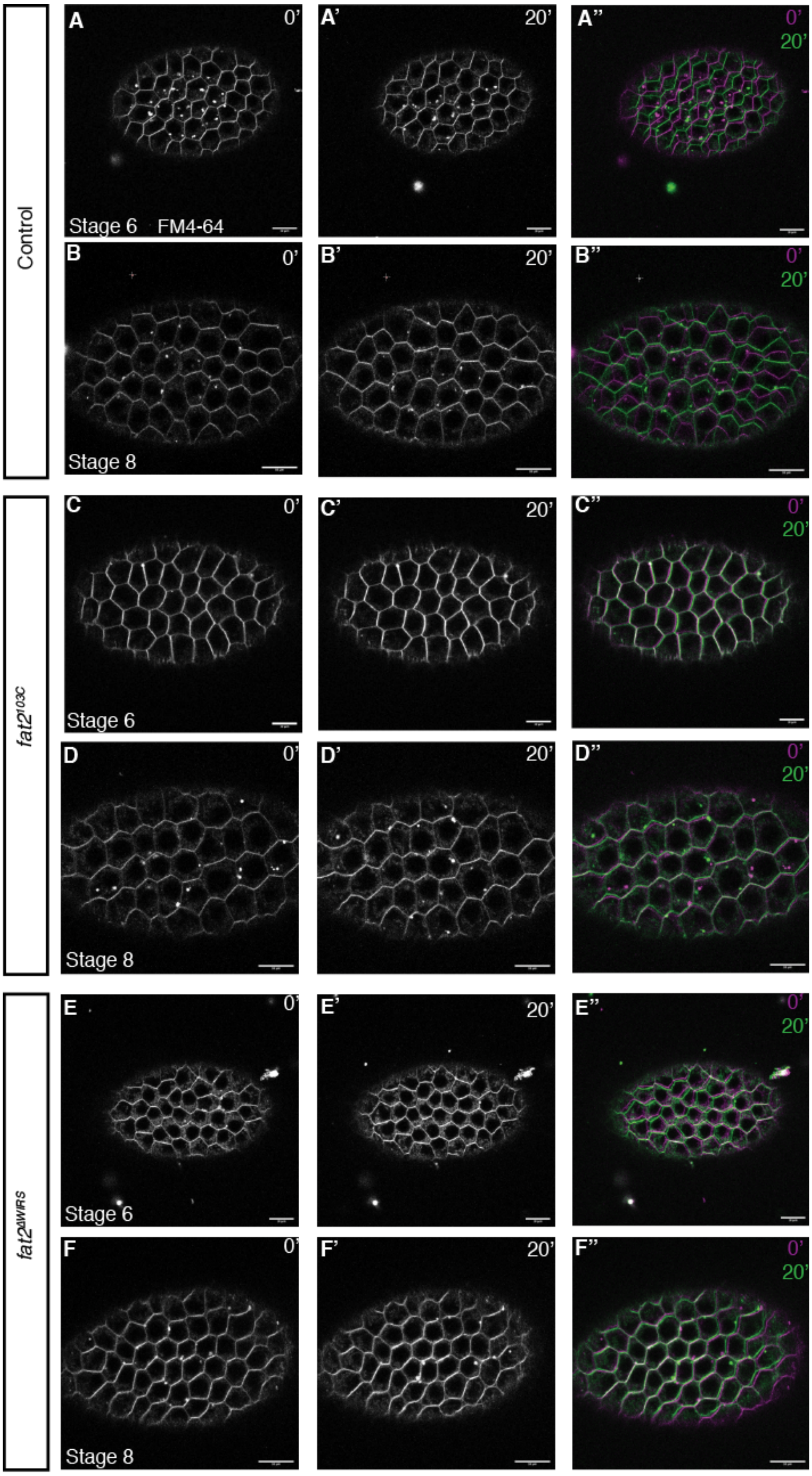

Fig. S2. WIRS motifs are required for *ex vivo* egg chamber rotation

(A-F) *Ex vivo* cultured egg chambers of indicated genotypes and stages shown at 0 min and 20 min of incubation. Right panels show overlays. Cell membranes are visualized by FM4-64. Scale bars: 10 μm.

Development • Supplementary information

Table S1. Oligonucleotides used for generation of *fat2*$^{\Delta WIRS}$ flies.

**Primer for the gRNAs**	
fat2$^{\Delta WIRS}$ -gRNA fwd	tgcagaacaagtcgtcgtacaagg
fat2$^{\Delta WIRS}$ -gRNA rev	aaacccttgtacgacgacttgttc
**Primers used for donor vector**	
fat2$^{\Delta WIRS}$ *LHA-PAM fwd*	taagcaggatcctcagcgtggcttcatcggttg
fat2$^{\Delta WIRS}$ *LHA-PAM rev*	gtgagttcaagttcgcttccttgtacg
fat2$^{\Delta WIRS}$ *PAM-WIRS1 fwd*	caagaacaagtcgtcgtacaaggaa
fat2$^{\Delta WIRS}$ *PAM-WIRS1 rev*	gcacattttccagttcatcgccggcgatgtctaagttatttacaaaattc
fat2$^{\Delta WIRS}$ *WIRS1-WIRS2 fwd*	gaattttgtaaataacttagacatcgccggcgatgaactggaaaatgtgc
fat2$^{\Delta WIRS}$ *WIRS1-WIRS2 rev*	ttgacttaccattgttcagtttattatgcatttgctcgcaccattcttgt
fat2$^{\Delta WIRS}$ *WIRS2-WIRS3 fwd*	acaagaatggtgcgagcaaatgcataataaactgaacaatggtaagtcaa
fat2$^{\Delta WIRS}$ *WIRS2-WIRS3 rev*	ttgatttatagtcttcattctcgattcttgaaagtggctgattacttgat
fat2$^{\Delta WIRS}$ *WIRS3-RHA fwd*	atcaagtaatcagccactttcaagaatcgagaatgaagactataaatcaa
fat2$^{\Delta WIRS}$ *WIRS3-RHA rev*	taagcaggatcctgctgttggatccaatttctccgac

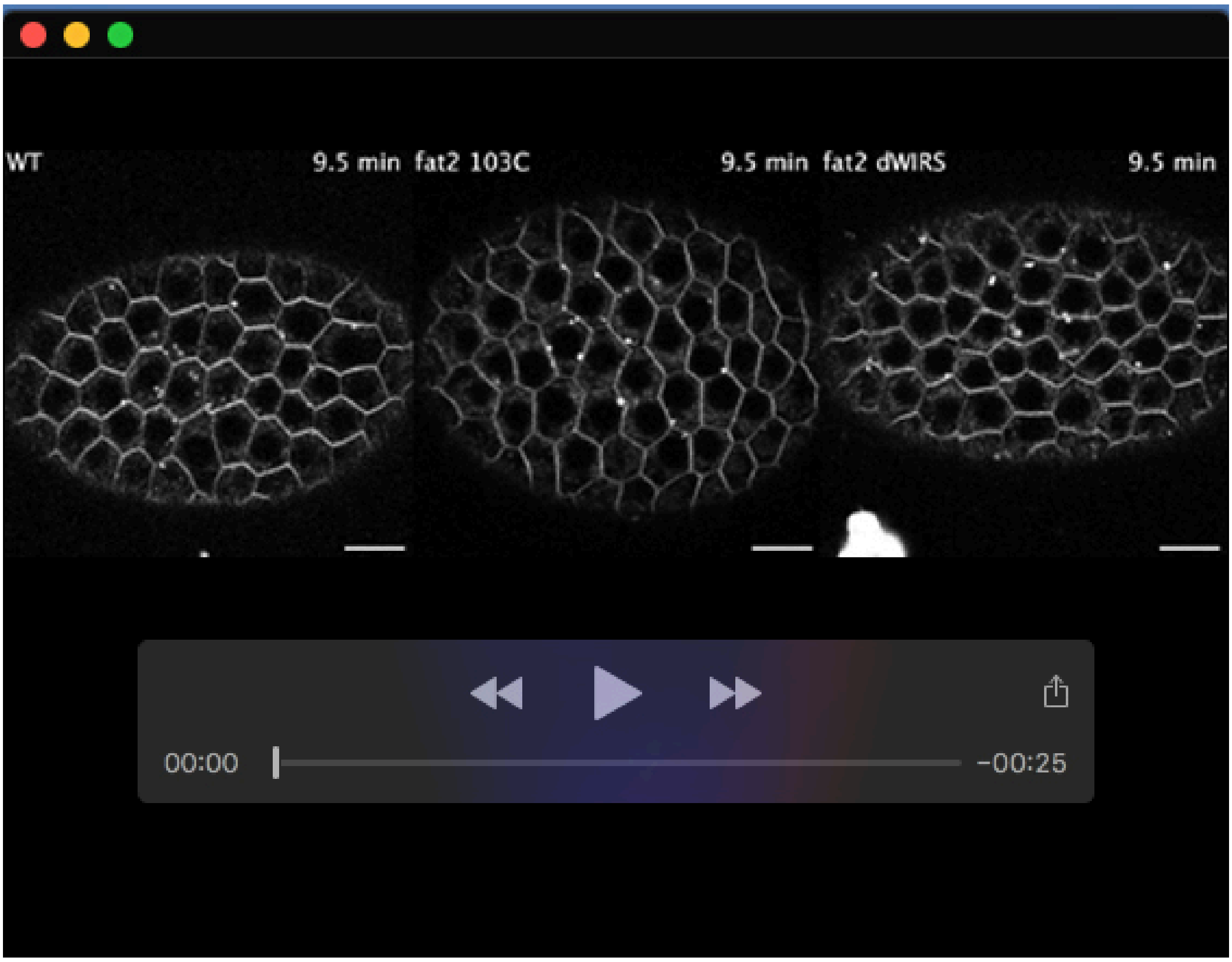

Movie 1. WIRS motifs are required for *ex vivo* egg chamber rotation

Time-lapse movies of *ex vivo* cultured egg chambers of indicated genotypes and stages. Cell membranes are visualized by FM4-64. Scale bars: 10 μm.

References

Chlasta, J., Milani, P., Runel, G., Duteyrat, J.L., Arias, L., Lamire, L.A., Boudaoud, A., and Grammont, M. (2017). Variations in basement membrane mechanics are linked to epithelial morphogenesis. Development *144*, 4350-4362.

Crest, J., Diz-Munoz, A., Chen, D.Y., Fletcher, D.A., and Bilder, D. (2017). Organ sculpting by patterned extracellular matrix stiffness. Elife *6*, eLife24958.

Töpfer, U., Guerra Santillan, K.Y., Fischer-Friedrich, E., and Dahmann, C. (2022). Distinct contributions of ECM proteins to basement membrane mechanical properties in *Drosophila*. Development *149*, dev200456.

Development • Supplementary information
