# Supplementary material for: The WIRS motifs in Fat2 are required for Drosophila egg chamber rotation but not for elongation
Source: Development. 2025 Jan 17;152(2):DEV204201. doi: 10.1242/dev.204201 (PMC11829772; doi:10.1242/dev.204201)
Supplement: Supplementary file 1 [file develop-152-204201_review_history.pdf]

The WIRS motifs in Fat2 are required for *Drosophila* egg chamber rotation but not for elongation

Akanksha Bhatt, Valentin Ruffine, Uwe Töpfer, Jinhee Ryu, Elisabeth Fischer-Friedrich and Christian Dahmann

DOI: 10.1242/dev.204201

Editor: Thomas Lecuit

Review timeline

Original submission:	20 June 2024
Editorial decision:	8 October 2024
First revision received:	5 December 2024
Accepted:	18 December 2024

Original submission

First decision letter

MS ID#: dev.204201

MS TITLE: The WIRS motifs in Fat2 are required for *Drosophila* egg chamber rotation but not elongation

AUTHORS: Akanksha Bhatt, Valentin Ruffine, Uwe Töpfer, Jinhee Ryu, Elisabeth Fischer-Friedrich and Christian Dahmann

I have now received all the referees reports on the above manuscript, and have reached a decision. The referees' comments are appended below, or you can access them online: please go to.

The overall evaluation is positive and we would like to publish a revised manuscript in Development, provided that the referees' comments can be satisfactorily addressed. Please attend to all of the reviewers' comments in your revised manuscript and detail them in your point-by-point response. If you do not agree with any of their criticisms or suggestions explain clearly why this is so. If it would be helpful, you are welcome to contact us to discuss your revision in greater detail. Please send us a point-by-point response indicating your plans for addressing the referees' comments, and we will look over this and provide further guidance.

Reviewer 1

Advance summary and potential significance to field

This is a very nice, focused paper, looking at the role of on Fat2 in Drosophila egg chamber rotation and elongation. Evidence has been published for a requirement for the Fat2 intracellular domain to interact with the WAVE regulatory complex during egg chamber elongation. Here, the authors directly address this requirement by precise deletion of the WRC interacting receptor sequence (WIRS) motifs in Fat2. The results are surprising, as deletion of the WIRS motifs blocks egg chamber rotation (as expected based on the published work), but this failure of rotation does not prevent egg chamber elongation - this reveals that previous models suggesting an essential role for rotation in elongation are wrong. There is also additional valuable characterization of the control vs fat2 vs Δ WIRS phenotype (i.e. effects on actin filaments, collagen fibers, basement membrane stiffness).

© 2024. Published by The Company of Biologists under the terms of the Creative Commons Attribution License (https://creativecommons.org/licenses/by/4.0/).

The manuscript is clear and well-written and the data convincing. Overall I recommend publication.

I only have some minor comments:
Lines 168-169: 'The stiffness of the stripes was increased by ~7%, similar to the control (Fig. 3F,G)' - I'm unsure how 7% was arrived at, and how I see that by looking at panels F and G? I suppose this is an average of the max intensity in the stripes? Similarly the next statement that the stripes are shorter? I realize that this made by hard to quantify, but if it's just an estimate/impression, then maybe be upfront about this?
On a point of clarification, for all the statistics, the figure legends include statements such as 'n=5 egg chambers for each genotype and stage'. I assume this means egg chambers from independent flies i.e. true biological replicates? Otherwise, if egg chambers are taken from the same fly, then we have no way of knowing whether that fly is typical of the population. Some more details in the relevant section of the Materials and Methods would be helpful.

Reviewer 2

Advance summary and potential significance to field

Bhatt at colleagues generate an allele of Drosophila Fat2 with its intracellular WIRS motifs specifically disrupted. Homozygous mutants are viable and fertile, allowing analyses of egg chamber morphogenesis. Remarkably, egg chamber elongation is unaffected, despite lack of egg chamber rotation, a behavior previously implicated as a driver of the elongation. This lack of rotation is shown in two distinct assays: one of egg chamber explants and one of egg chambers in vivo. Both basal actin filaments of the follicle cells and collagen IV fibrils of the basement membrane display a regular degree of planar polarity, but abnormalities in organization as well. Globally, the mutant egg chambers display greater ECM stiffness at their centers versus their poles, similar to controls, implicating polar relaxation as an alternate mechanism of elongation. Overall, this paper seems to clarify that egg chamber rotation is not required for egg chamber elongation, and shows that the WIRS motifs in Fat2 are functional and required for the normal rotation process. The manuscript should be of interest to readers of Development. However, the points below should be addressed.

Comments for the author

1. Referring to the literature, the authors state that egg chamber rotation occurs during stages 1 - 8, but it is unclear whether their analyses of fat2[deltaWIRS] rules out rotation at early stages. Their analyses of explants showed no rotation at stages 6 and 8. It wasn't clear whether the in vivo "matrix-labeling technique for real-time and inferred location" would be able to detect whether rotation occurred at early stages or not—this should be better explained or tested to conclusively rule out a requirement for rotation.

2. The recent conflicting reports of whether egg chamber elongation can occur with egg chamber rotation, or not, should be more clearly presented in the Introduction so the motivation/advance of the current paper is clearer. The reports are: Chen et al (2017) "A Cell Migration Tracking Tool Supports Coupling of Tissue Rotation to Elongation"; and Aurich and Dahmann (2016) "A mutation in fat2 uncouples tissue elongation from global tissue rotation."

3. In the Discussion, it would be helpful to add a brief speculation about why deletion of the full Fat2 cytoplasmic tail allows rotation in vivo (Chen et al, 2017), whereas deletion of the WIRS motifs within the tail prevents the rotation. Or state that the reason for the difference remains unknown.

4. It is stated that "The topography of egg chambers of fat2[103C] or fat2[deltaWIRS] homozygous mutant flies resembled that of control egg chambers." However, the topography image of the fat2[deltaWIRS] mutant seems distinct from the other two, apparently with more distinctive planar polarized ridges (compare Fig 3D to Fig 3C and E). Direct quantifications of the topography data represented by the images in Fig 3C-E seems warranted. Also, a quantification of "Stiffness-height correlation" is only provided for control samples (see Fig 3E). Extending the assessment of the "Stiffness-height correlation" to the two mutants would be another way to more rigorously test the egg chamber topography of fat2[deltaWIRS] mutants.

© 2024. Published by The Company of Biologists under the terms of the Creative Commons Attribution License (https://creativecommons.org/licenses/by/4.0/).

5. Can a statistical test be applied to the "Center-to-pole stiffness ratio" data in Fig 3O?

6. Since deletion of the WIRS motifs is expected to affect recruitment of the WAVE regulatory complex (WRC) and downstream actin polymerization, greater assessment of basal actin protrusions is warranted. It is stated that "basal actin-rich protrusions of follicle cells were not properly polarized in follicle cells of fat2[deltaWIRS] homozygous mutant egg chambers compared with controls (Fig. 4L,M)". In some locations, the protrusions are abnormally present, but in other tissue regions, planar polarity of the protrusions is apparent (see Fig 4M). Assessments of these data should be expanded.

7. Also, the expected effect of deleting the WIRS motifs Fat2 on localization of the WRC should be discussed. In particular, this point should be discussed in relation to Williams et al (2022) "Fat2 polarizes the WAVE complex in trans to align cell protrusions for collective migration." This analysis indicated that there is relatively little WRC accumulation on the side of the cell-cell contact with the Fat2 cytoplasmic tail, in contrast to the strong accumulation of the WRC on the opposite side of the cell-cell contact.

8. Can the authors add some speculation about how the planar polarization of basal F-actin and basement collagen IV fibrils arises in fat2[deltaWIRS] homozygous mutant egg chambers in the absence of egg chamber rotation? Or state that this remains unknown.

Minor issues:

There are orange plus symbols on the graphs in Fig 3K and 3M, and they aren't mentioned in the legend.

In all two-color images, the combination of red and green should be avoided since people with color-blindness can't distinguish these colors. Using purple (R+B) instead of red is a better option in combination with green.

First revision

Author response to reviewers' comments

We thank the reviewers for their thoughtful and constructive comments that helped us to improve the manuscript.

Reviewer 1: This is a very nice, focused paper, looking at the role of on Fat2 in Drosophila egg chamber rotation and elongation. Evidence has been published for a requirement for the Fat2 intracellular domain to interact with the WAVE regulatory complex during egg chamber elongation. Here, the authors directly address this requirement by precise deletion of the WRC interacting receptor sequence (WIRS) motifs in Fat2. The results are surprising, as deletion of the WIRS motifs blocks egg chamber rotation (as expected based on the published work), but this failure of rotation does not prevent egg chamber elongation - this reveals that previous models suggesting an essential role for rotation in elongation are wrong. There is also additional valuable characterization of the control vs fat2 vs ΔWIRS phenotype (i.e. effects on actin filaments, collagen fibers, basement membrane stiffness).
The manuscript is clear and well-written and the data convincing. Overall I recommend publication.

I only have some minor comments:
Lines 168-169: 'The stiffness of the stripes was increased by ~7%, similar to the control (Fig. 3F,G)' - I'm unsure how 7% was arrived at, and how I see that by looking at panels F and G? I suppose this is an average of the max intensity in the stripes? Similarly the next statement that the stripes are shorter? I realize that this made by hard to quantify, but if it's just an estimate/impression, then maybe be upfront about this?

© 2024. Published by The Company of Biologists under the terms of the Creative Commons Attribution License (https://creativecommons.org/licenses/by/4.0/).

OUR RESPONSE: We clarified that the 7% increase relates to the stiffness in the bulk versus stripe region. We derived this value by comparing the Young's modulus in the stiffness maps of control and fat2[deltaWIRS] egg chambers (Fig. 3F,G).
As the reviewer pointed out, the length of the stripes is difficult to quantify. We therefore estimated and compared the length of the stripes based on visual inspection of the images. We refer to this qualitative assessment as "appeared to be shorter".

On a point of clarification, for all the statistics, the figure legends include statements such as 'n=5 egg chambers for each genotype and stage'. I assume this means egg chambers from independent flies i.e. true biological replicates? Otherwise, if egg chambers are taken from the same fly, then we have no way of knowing whether that fly is typical of the population. Some more details in the relevant section of the Materials and Methods would be helpful.
OUR RESPONSE: We added to the Materials and Methods section the sentence: "The number of replicates refers to egg chambers isolated from different individual flies."

Reviewer 2: SUMMARY OF THE ADVANCE MADE IN THIS PAPER AND ITS POTENTIAL SIGNIFICANCE TO THE FIELD
Bhatt at colleagues generate an allele of Drosophila Fat2 with its intracellular WIRS motifs specifically disrupted. Homozygous mutants are viable and fertile, allowing analyses of egg chamber morphogenesis. Remarkably, egg chamber elongation is unaffected, despite lack of egg chamber rotation, a behavior previously implicated as a driver of the elongation. This lack of rotation is shown in two distinct assays: one of egg chamber explants and one of egg chambers in vivo. Both basal actin filaments of the follicle cells and collagen IV fibrils of the basement membrane display a regular degree of planar polarity, but abnormalities in organization as well. Globally, the mutant egg chambers display greater ECM stiffness at their centers versus their poles, similar to controls, implicating polar relaxation as an alternate mechanism of elongation. Overall, this paper seems to clarify that egg chamber rotation is not required for egg chamber elongation, and shows that the WIRS motifs in Fat2 are functional and required for the normal rotation process. The manuscript should be of interest to readers of Development. However, the points below should be addressed.

SUGGESTIONS TO AUTHORS
1. Referring to the literature, the authors state that egg chamber rotation occurs during stages 1 - 8, but it is unclear whether their analyses of fat2[deltaWIRS] rules out rotation at early stages. Their analyses of explants showed no rotation at stages 6 and 8. It wasn't clear whether the in vivo "matrix-labeling technique for real-time and inferred location" would be able to detect whether rotation occurred at early stages or not—this should be better explained or tested to conclusively rule out a requirement for rotation.
OUR RESPONSE: We clarified that M-TRAIL assays egg chamber rotation between the time point at which GFP-Cg25C secretion upon clone induction begins and the time of analysis. We generated clones up to 48 h before analysis of stage 9 egg chambers. Cg25C secretion starts 6 h after clone induction (Chen et al. 2017). GFP-Cg25c secretion will therefore start ~42 h before analysis in stage 9, which corresponds to stage 2-3. Thus, our analysis rules out egg chamber rotation of the fat2[deltaWIRS] mutant at early stages (from stage 2-3 onwards). We revised the manuscript as follows (lines 222-228): "The angular velocity of trail deposition is a measure of egg chamber rotation that takes place between the beginning of GFP-Cg25c secretion upon clone induction and the time of analysis. We generated clones secreting GFP-Cg25c and analyzed stage 9 egg chambers 10 h, 13 h, 24h, or 48 h later for the presence of trails. Based on the duration of egg chamber development (Spradling, 1993) and the time required for GFP-Cg25C secretion (Chen et al. 2017), we expected that the analysis of egg chambers 48 h after clone induction assayed rotation from stage 2-3 onwards."

2. The recent conflicting reports of whether egg chamber elongation can occur with egg chamber rotation, or not, should be more clearly presented in the Introduction so the motivation/advance of the current paper is clearer. The reports are: Chen et al (2017) "A Cell Migration Tracking Tool Supports Coupling of Tissue Rotation to Elongation"; and Aurich and Dahmann (2016) "A mutation in fat2 uncouples tissue elongation from global tissue rotation."

© 2024. Published by The Company of Biologists under the terms of the Creative Commons Attribution License (https://creativecommons.org/licenses/by/4.0/).

OUR RESPONSE: We added the following sentence to the Introduction: “However, a recent study indicated that ECM fiber-like structures can align and egg chambers can elongate in the absence of egg chamber rotation (Aurich and Dahmann, 2016).”

3. In the Discussion, it would be helpful to add a brief speculation about why deletion of the full Fat2 cytoplasmic tail allows rotation in vivo (Chen et al, 2017), whereas deletion of the WIRS motifs within the tail prevents the rotation. Or state that the reason for the difference remains unknown.
OUR RESPONSE: We now state that the reason for this difference is unknown.

4. It is stated that "The topography of egg chambers of fat2[103C] or fat2[deltaWIRS] homozygous mutant flies resembled that of control egg chambers." However, the topography image of the fat2[deltaWIRS] mutant seems distinct from the other two, apparently with more distinctive planar polarized ridges (compare Fig 3D to Fig 3C and E). Direct quantifications of the topography data represented by the images in Fig 3C-E seems warranted. Also, a quantification of "Stiffness-height correlation" is only provided for control samples (see Fig 3E). Extending the assessment of the "Stiffness-height correlation" to the two mutants would be another way to more rigorously test the egg chamber topography of fat2[deltaWIRS] mutants.
OUR RESPONSE: We now quantified the topography data by applying Fourier analysis (new Figure S1A). Fourier analysis is used here to identify and quantify spatial patterns in the topography data. We describe our result as follows (lines154-157):” Fourier analysis along the anteroposterior axis (see Materials and Methods) showed that the topography of egg chambers of fat2[103C] or fat2[deltaWIRS] homozygous mutant flies was smoother compared with the topography of control egg chambers (Fig. 3D,E, Fig. S1A), indicating differences in the structure of the basement membranes”. We also adjusted the (arbitrary) absolute height (z=0) in Fig. 3D to make this panel more comparable to panels C and E of Fig. 3. This change affects the visual perception of the topography (less purple color), but does not change the data itself, since the topography maps in Fig. 3 C-E only show relative heights.

We now also quantified the “Stiffness-height correlation” for egg chambers of the fat2[103C] and fat2[deltaWIRS] homozygous mutant flies (new Fig. 3L). Stiffness and height did not correlate. We also re-analyzed the “Stiffness-height correlation” for control egg chambers and now show that stiffness and height negatively correlate (new Fig. 3L). We describe this new result as follows (lines 157-162): “The topography negatively correlated with the spatial pattern of stiffness in control egg chambers (Fig. 3C.F,L), suggesting the presence of soft elevated structures. However, stiffness and topography did not correlate in egg chambers of fat2[103C] or fat2[deltaWIRS] homozygous mutant flies (Fig. 3D,E,G,H,L), indicating that such soft elevated structures are not present in the basement membrane of these mutants.”

5. Can a statistical test be applied to the "Center-to-pole stiffness ratio" data in Fig 3O?
OUR RESPONSE: We now applied a statistical test to the "Center-to-pole stiffness ratio", testing the significance of the difference of the stiffness ratio to 1. This analysis revealed a high significance for the control ($p<0.05$), a p-value of 0.063 for the fat2[deltaWIRS] mutant egg chamber and no significant difference for the fat2[103C] mutant (new Fig. 3O).

6. Since deletion of the WIRS motifs is expected to affect recruitment of the WAVE regulatory complex (WRC) and downstream actin polymerization, greater assessment of basal actin protrusions is warranted. It is stated that "basal actin-rich protrusions of follicle cells were not properly polarized in follicle cells of fat2[deltaWIRS] homozygous mutant egg chambers compared with controls (Fig. 4L,M)". In some locations, the protrusions are abnormally present, but in other tissue regions, planar polarity of the protrusions is apparent (see Fig 4M). Assessments of these data should be expanded.
OUR RESPONSE: We have now quantified the orientation of basal actin-rich protrusions (new panels Fig. 4O and P). We show that a significantly lower fraction of basal actin-rich protrusions is oriented perpendicular to the anteroposterior axis of the egg chamber in fat2[deltaWIRS] homozygous mutant egg chambers compared with controls.

7. Also, the expected effect of deleting the WIRS motifs Fat2 on localization of the WRC should be discussed. In particular, this point should be discussed in relation to Williams et al (2022) "Fat2 polarizes the WAVE complex in trans to align cell protrusions for collective migration." This analysis

© 2024. Published by The Company of Biologists under the terms of the Creative Commons Attribution License (https://creativecommons.org/licenses/by/4.0/).

indicated that there is relatively little WRC accumulation on the side of the cell-cell contact with the Fat2 cytoplasmic tail, in contrast to the strong accumulation of the WRC on the opposite side of the cell-cell contact.
OUR RESPONSE: We state that "[Fat2] is required *in trans* to enrich […] the WAVE regulatory complex (WRC) to the leading edge of the following cells" citing Williams et al. 2022) (lines 71-73). We also mention that based on biochemical data the binding of Fat2 in cis to the WRC plays a role in egg chamber rotation (Squarr et al., 2016) (lines 77-83). We discuss that "Our work now provides *in vivo* evidence that Fat2 acts *in cis* with the WAVE complex to direct follicle cell migration". (lines 265-266)

8. Can the authors add some speculation about how the planar polarization of basal F-actin and basement collagen IV fibrils arises in fat2[deltaWIRS] homozygous mutant egg chambers in the absence of egg chamber rotation? Or state that this remains unknown.
OUR RESPONSE: We state that "How actin filaments and Collagen IV fiber-like structures are aligned in the absence of egg chamber rotation needs to be revealed in the future". (lines 255-257)

Minor issues:

There are orange plus symbols on the graphs in Fig 3K and 3M, and they aren't mentioned in the legend.
OUR RESPONSE: Outliers are indicated (in this case by orange plus symbols), as is standard for box plots.

In all two-color images, the combination of red and green should be avoided since people with color-blindness can't distinguish these colors. Using purple (R+B) instead of red is a better option in combination with green.
OUR RESPONSE: Thank you for pointing this out to us. We have changed the color of the red/green images to purple/green (Fig. 1B-D, Fig. S2).

Second decision letter

MS ID#: dev.204201R1

MS TITLE: The WIRS motifs in Fat2 are required for *Drosophila* egg chamber rotation but not elongation

AUTHORS: Akanksha Bhatt, Valentin Ruffine, Uwe Töpfer, Jinhee Ryu, Elisabeth Fischer-Friedrich and Christian Dahmann

I have looked carefully at the revised manuscript and the responses to the reviewers comments. And in light of this I am happy to tell you that your manuscript has been accepted for publication in Development, pending our standard publication integrity checks.

© 2024. Published by The Company of Biologists under the terms of the Creative Commons Attribution License (https://creativecommons.org/licenses/by/4.0/).
